# Biochemical and morphological comparison of two tumour-cell-aggregation factors from rat ascites hepatoma cells.

**DOI:** 10.1038/bjc.1978.82

**Published:** 1978-04

**Authors:** Y. Hanaoka, K. Kudo, Y. Ishimaru, H. Hayashi

## Abstract

**Images:**


*Br. J. Cancer* (1978) **37**, 536

### BIOCHEMICAL AND MORPHOLOGICAL COMPARISON OF TWO TUMOUR-CELL-AGGREGATION FACTORS FROM RAT ASCITES HEPATOMA CELLS*

Y. HANAOKA, K. KUDO, Y. ISHIMARU AND H. HAYASHI

*From the Department of Pathology, Kumamoto University Medical School, Kumamoto 860, Japan*

Received 3 October 1977 Accepted 15 December 1977

**Summary.—Two tumour-cell-aggregation factors, derived from rat ascites hepatoma cells, had different antigenicity; one was not absorbed by immunoadsorbent chromatography with anti-rat serum antibody and the other was. Their activities were both lost by digestion with trypsin, but remained unchanged by oxidation with periodate, suggesting the role of the protein portions in their molecules. The potency of the unabsorbed factor was inhibited specifically by α-methyl-D-mannoside or D-mannose, while that of the absorbed factor was inhibited specifically by N-acetyl-D-glucosamine, suggesting that these carbohydrates may be concerned with the respective receptor structures at the tumour-cell surface. The unabsorbed factor induced not only cell aggregation (as shown in the form of simple apposition) but also cell adhesiveness characterized by development of intermediate junctions, desmosomes and tight junctions, while the absorbed factor produced only simple apposition, suggesting their functional difference.**

As previously described (Kudo *et al.*, 1974; Kudo *et al.*, 1976), two glycoproteins capable of inducing tumour-cell-aggregation have been separated from rat ascites hepatoma cells forming cell islands *in vivo.* They had different antigenicity; one, with a strong potency, was not absorbed by immunoadsorbent chromatography with anti-rat serum antibody, while the other, with a weak potency, was. As became well known after the introduction of Landsteiner's hapten inhibition techniques, many haemagglutinins react with specific carbohydrates on the erythrocyte surface (Burger and Goldberg, 1967; Inbar and Sachs, 1969; Lis *et al.*, 1970). These studies have raised the possibility that carbohydrates may play an important part in the interaction between lectins and the mammalian cell surface (Aub *et al.*, 1965; Burger, 1969; Biddle *et al.*, 1970). Accordingly, it would be of interest to investigate whether these factors may react with specific carbohydrates.

As reported earlier (Ishimaru *et al.*, 1975; Ishimaru *et al.*, 1976), a mixed preparation of these 2 factors caused both aggregation (as shown in the form of simple apposition) of rat ascites hepatoma AH109A cells present in a free form *in vivo,* and adhesiveness of the cells, characterized by gradual development of well-defined junctional complexes (including intermediate junctions, desmosomes and focal tight junctions) during a 24 h incubation. Accordingly, it would be of interest to clarify which type of these factors may be associated with development of junctional complexes mentioned above.

### MATERIALS AND METHODS

*Rat ascites hepatoma.*—Rat ascites hepatomas AH136B (forming cell islands *in vivo*)

* No. 6 of the studies on tumour-cell aggregation-promoting factors.

Correspondence to: Professor H. Hayashi, Department of Pathology, Kumamoto University Medical School, Kumamoto 860, Japan.

and AH109A (present as free cells *in vivo*) have been maintained in our laboratory by routine 10-day passage of $10^6$ AH136B cells, and by routine weekly passage of $2\times10^6$ AH109A cells injected i.p. into 80–100 g male rats of the Donryu strain (Kudo *et al.*, 1976).

*Isolation of aggregation-promoting factor (APF).*—This was performed by the method previously described by Kudo *et al.* (1976). APF was released from $15\times10^8$ AH136B cells, suspended in Hanks' balanced salt solution (HBS) free of calcium and magnesium in the cold, by 50 gentle pipettings and eluted on DEAE-Sephadex and Bio-gel. The active fraction was eluted through an immunoadsorbent column with rabbit anti-rat serum antibody in 0·02M phosphate buffer (pH 6·8) followed by 1·0M acetic acid (pH 2·4). The first component was used as the unabsorbed APF after re-chromatography under the same condition as noted above. The second component was used as the absorbed APF. Estimation of protein concentration was done by the method of Lowry *et al.* (1951) using bovine serum albumin as a standard.

*In vitro assay for cell aggregation.*—For carbohydrate inhibition experiments, equal volumes (1·0 ml) of the unabsorbed APF (100 μg/ml) or the absorbed APF (500 μg/ml) and of AH109A cell suspension ($1\cdot5\times10^6$/ml in HBS free of glucose) were mixed in a Falcon tube ($1\cdot5\times9\cdot5$ cm) and incubated at 37°C in a roller tube culture apparatus, model Te-Her (Hirasawa Co., Tokyo, Japan) run at 1 rotation/8 min (Kudo *et al.*, 1976). These APFs respectively induced cell aggregation graded+ at each concentration indicated; over 50% of the originally suspended cells were aggregated after 30 min of incubation.

For electronmicroscope experiments, AH109A cells were finally suspended at a concentration of $2\times10^6$ cells/ml in Earle's MEM containing 20% normal rat serum. Each APF was tested at the same concentrations as noted above. At 24 h after addition of these APFs, cell aggregates formed were removed for electronmicroscopic examination, following the method previously described (Ishimaru *et al.*, 1975).

*Carbohydrate inhibition experiments.*—D-galactose D-glucose, and N-acetyl-D-glucosamine (Wako Chemical Co., Osaka, Japan), β-methyl-D-glucoside and N-acetyl-D-mannoside (Nakarai Chemical Co., Kyoto, Japan), amine D-mannose (Katayama Chemical Co., Osaka, Japan), N-acetyl-D-galactosamine (Seikagaku Kogyo Co., Tokyo, Japan), α-methyl-D-glucoside (Fluka AG, Buchs SG, Switzerland) and α-methyl-D-mannoside (Sigma, St Louis, Missouri, USA) were used. These sugar preparations were dissolved at various concentrations (25, 50, 100, 200, 300, and 400 mM) in HBS free of glucose. Ovomucoid (Sigma, St Louis, Missouri, USA) was dissolved at various concentrations (25, 50, 100, and 200 μg/ml) in HBS free of glucose. Equal volumes (1·0 ml) of APF solution and sugar solution were mixed and then allowed to stand at 37°C for 30 min. After dialysis against HBS free of glucose at 4°C for 12 h to remove free sugars, the mixture solution (1·0 ml) was tested for cell aggregation.

*Preparation of specific affinity adsorbents.*—D-mannose-starch affinity adsorbent was prepared by the method of Matsumoto and Osawa (1972) using finely powdered corn starch and D-mannose. It has been known that D-mannose is cross-linked to starch in the resulting derivative. Ovomucoid-Sepharose 4B adsorbent was prepared according to the method of Porath *et al.* (1967).

*Treatment of APF with trypsin and periodate.*—Trypsin (Boehringer Mannheim GmbH, West Germany) was dissolved at various concentrations (25, 50, and 100 μg/ml in Hepes-buffered saline, pH 7·4). According to the method of Pessac and Defendi (1972) equal volumes (1·0 ml) of the unabsorbed APF (100 μg/ml) or the absorbed APF (500 μg/ml) and of trypsin solution were mixed and incubated for 3 h at 37°C, and then each reaction mixture was heated at 85°C for 30 min to inactivate the proteolytic activity. The potency of the unabsorbed and absorbed APFs remained unchanged by such heating (Kudo *et al.*, 1974, 1976). After centrifugation at 10,000 g for 30 min, each supernatant was assayed for cell aggregation.

Periodate (Katayama Chemical Co., Osaka, Japan) was dissolved at various concentrations (1, 5, and 10 mM in distilled water). Equal volumes (1·0 ml) of the unabsorbed APF (100 μg/ml) or the absorbed APF (500 μg/ml) and of periodate solution were mixed and incubated for 24 h at 4°C by the method of Pessac and Defendi (1971). After dialysis against HBS free of glucose at 4°C for 12 h, to remove free periodate, each reaction mixture was tested for cell aggregation.

### RESULTS

### *Carbohydrate inhibition experiments*

Percentage inhibition was calculated from the expression $\frac{A-B}{A} \times 100$, where A is the number of aggregating cells in the absence of sugars, while B is the number in the presence of sugars. Inhibition curves were constructed by plotting the calculated values for percentage inhibition *vs* the common logarithm of mM of sugars added. α-methyl-D-mannoside and D-mannose strongly inhibited tumour-cell aggregation by the unabsorbed APF; at a concentration of 100 mM, these sugars resulted in an almost complete inhibition of cell aggregation (Fig. 1). The effects of other sugars tested were apparently less marked or negligible. On the other hand, the potency of the absorbed APF was strongly inhibited by N-acetyl-D-glucosamine (Fig. 2); at a concentration of 100 mM, this sugar resulted in an almost complete inhibition of cell aggregation. No or little inhibition of cell aggregation by the absorbed APF was detected with other sugars. Furthermore, ovomucoid, which is known as an N-acetyl-D-glucosamine-rich glycoprotein in its oligosaccharide chain, also resulted in the complete inhibition of cell aggregation by the absorbed APF, when tested at a concentration of 100 μg/ml.

FIG. 1.—Inhibition by various monosaccharides of tumour-cell aggregation by unabsorbed APF. % inhibition was calculated from the expression $\frac{A-B}{A} \times 100$, where A is the number of aggregating cells in the absence of sugars, while B is the number in the presence of sugars. Inhibition curves were constructed by plotting the calculated values for percentage inhibition *vs* the common logarithm of mM of sugars added. ○, α-methyl-D-mannoside. ●, D-mannose. ▽, α-methyl-D-glucoside. ▼, β-methyl-D-glucoside. ×, N-acetyl-D-mannosamine. □, D-glucose. ■, D-galactose. △, N-acetyl-D-glucosamine. ▲, N-acetyl-D-galactosamine.

FIG. 2.—Inhibition by various monosaccharides of tumour-cell aggregation by absorbed APF. % inhibition was calculated as in Fig. 1. ○, α-methyl-D-mannoside. ●, D-mannose. ▽, α-methyl-D-glucoside. ▼, β-methyl-D-glucoside. ×, N-acetyl-D-mannosamine. □, D-glucose. ■, D-galactose. △, N-acetyl-D-glucosamine. ▲, N-acetyl-D-galactosamine.

### *Sugar-specific affinity adsorbent chromatography*

(*a*) *D-mannose-starch adsorbent chromatography of unabsorbed APF.*—After concentration under vacuum pressure dialysis, 5 ml of the unabsorbed APF (1–2 mg/ml in 0·02 M phosphate buffer, pH 6·8) were

FIG. 3.—D-mannose-starch adsorbent chromatography of unabsorbed APF. Elution was performed with 0·02M phosphate buffer (pH 6·8) and then with 1·0M acetic acid (pH 2·4). Flow rate was 18 ml/h. Effluent was collected in 5g fractions. Each fraction was tested at the same concentration (absorbency 0·5 at 280 nm/ml) for aggregation activity.

FIG. 4.—Ovomucoid-Sepharose 4B adsorbent chromatography of absorbed APF. Elution was performed with 0·02 M phosphate buffer followed by 1·0 M acetic acid. Flow rate was 18 ml/h and 5g fractions were collected. Each fraction was tested at the same concentration (absorbency 0·5 at 280 nm/ml) for aggregation activity.

eluted on a column (2·0 × 8·0 cm) of D-mannose-starch gel equilibrated with the same buffer. Elution was done with 0·02 M phosphate buffer (pH 6·8) followed by 1·0 M acetic acid (pH 2·4). Flow rate was 18 ml/h and 5 g effluent fractions were collected. Total yield was about 99% of the applied samples, measured as the absorbency at 280 nm; the first comprising 83% and the second, 16%. The second (absorbed) component was apparently more potent than the first (unabsorbed) component for cell aggregation (Fig. 3); it was active in 32-fold dilution. In order to avoid a problem due to column overloading, the first (unabsorbed) component was re-chromatographed under the same condition as noted above. The recovery of the applied sample was about 99%; the first comprising 91% and the second, 8%. The second (absorbed) component was clearly potent, and active in 32 fold dilution, while the first (unabsorbed) component was inactive. The evidence showing that the unabsorbed APF is effectively retained by the column and then eluted without loss of its activity in an acid condition seemed to re-confirm the specific inhibition by D-mannose of the unabsorbed APF.

(*b*) *Ovomucoid-Sepharose 4B adsorbent chromatography of absorbed APF.*—Chromatography with ovomucoid-Sepharose 4B adsorbent derivative was done for the absorbed APF. Before the chromatography, 5 ml of the absorbed APF (1–2 mg/ml), dialysed against 0·02M phosphate buffer (pH 6·8) for 12 h at 4°C and then concentrated, were eluted on D-mannose starch gel as described above, because ovomucoid sample has been known to contain a small quantity of D-mannose in the oligosaccharide chain. Total yield was about 100% of the applied samples, measured as the absorbency at 280 nm; the first comprising 89% and the second, 11%. The first (unabsorbed) component was potent for cell aggregation corresponding to the absorbed APF potency, while the second (absorbed) component was inactive, indicating that absorption by D-mannose in the ovomucoid sample of the absorbed APF is negligible, if indeed it exists. Accordingly, 5 ml of the above first (unabsorbed) component (1–2 mg/ml in 0·02M phosphate buffer, pH 6·8) was applied to an ovomucoid-Sepharose 4B affinity adsorbent column (2·0 × 8·0 cm). Elution was done with 0·02 M phosphate buffer (pH 6·8) followed by 1·0M acetic acid (pH 2·4). Flow rate was 18 ml/h, and 5 g effluent fractions were collected. Total yield was about 98% of the applied sample, measured as the adsorbency at 280 nm; the first comprising 38% and the second, 60% (Fig. 4); the activity was found only in the second (absorbed) component. Its activity became positive in 6-fold dilution with HBS after chromatography. The observations seemed to reconfirm the specific inhibition of the absorbed APF by N-acetyl-D-glucosamine (but not by D-mannose).

*Treatment with trypsin and periodate*

The potency of the absorbed and unabsorbed APFs was completely abolished by treatment with trypsin, even at low concentration (25 μg/ml), aggregating cells were rarely found after 30 min of observation. On the other hand, the activity of these APFs remained unchanged by treatment with periodate at different concentrations (1, 5, and 10 mM). It was suggested that the potency of these APFs is similarly resistant to periodate, but sensitive to trypsin.

FIG. 5.—Adherent AH109A cells observed after 24h incubation with the unabsorbed APF. The adhesiveness of these cells is clearly close and characteristic. The cell-surface regions showing close contact are prominent. Such an electronmicroscopic picture closely resembles that induced by a mixture of the unabsorbed and absorbed APFs, as previously reported. S→, simple apposition. I→, intermediate junction. D→, desmosome. F→, focal tight junction. ×5000.

(a) (b) (c) (d)

FIG. 6.—(a) Simple apposition observed in adherent AH109A cells in 24h incubation with unabsorbed APF. Two plasma membranes are separated by an intercellular space of about 30 nm. × 30,000. (b) Intermediate junction in adherent cells, consisting of outer leaflets disposed in a parallel fashion and separated by a space of 10–20 nm with low electron density. In the cytoplasm subjacent to the inner leaflet, electron-dense materials are seen. × 68,000. (c) Desmosome in adherent cells, characterized by one distinct laminar plaque. Prominent endoplasmic fibrils are related to the plaque. × 54,000. (d) Focal tight junction in adherent cells, characterized by punctate fusion of outer leaflets. × 68,000.

*Electronmicroscopic examination*

(*a*) *Effect of unabsorbed APF*.—After 30 min incubation, AH109A cell aggregates settled to the bottom of the Falcon tubes. The majority of the aggregated cells after 24 h incubation could not be dissociated by pipetting. Microscopically, the aggregated cells showed a tendency to arrange themselves in a concentric pattern.

The cell aggregates, formed in 24 h incubation, were examined by the electronmicroscope. In addition to simple apposition as described by Farquhar and Palade (1963), cell contact was characterized by development of well defined tripartite junctional complexes (Figs. 5 and 6). The intermediate junctions consisted of 2 outer leaflets disposed in a parallel fashion and separated by an intercellular space of less than 20 nm, occupied by homogeneous materials of low density which resemble those described by Farquhar and Palade (1963). The cytoplasm subjacent to the inner leaflets showed moderate electron density. The desmosomes observed consisted of 2 outer leaflets running in a parallel fashion and separated by an intercellular space of about 20 nm, containing a central disc of electron-dense materials. In the cytoplasm subjacent to each inner leaflet, there was one distinct laminar plaque running parallel to each inner leaflet, accompanied by prominent endoplasmic fibrils, which resemble those reported by Farquhar and Palade (1963), Trelstad *et al.* (1967) and Lentz and Trinkaus (1971). The focal tight junctions, as described by Trelstad *et al.* (1967), were characterized by a narrow gap less than 4 nm in width, which was formed by close approximation of outer leaflets and punctate fusion of outer leaflets. The frequency of simple apposition, intermediate junction, desmosome and focal tight junction observed at this stage was in the ratio of 10:7:4:0·2 respectively when counted for 50 cells. These electronmicroscope pictures of cell adhesiveness were essentially indistinguishable from those revealed at 24 h after contact with a mixed preparation of the unabsorbed and absorbed APFs (Ishimaru *et al.*, 1975, 1976).

(*b*) *Effect of absorbed APF*.—The macroscopic AH109A cell aggregates, formed in 24 h incubation, resembled those seen in the above cultures, though the aggregated cells were mostly dissociated by pipetting. The intercellular spaces of the aggregated cells were larger and areas of cellular apposition were smaller; cell-surface regions showing close contact were rare. The areas of close contact consisted only of simple apposition-like structures of plasma membranes. Apposed plasma membranes were separated by a space of 20–30 nm with no electron density. No structures resembling intermediate junctions, desmosomes or focal tight junctions were observed.

### DISCUSSION

The present observations suggest that the tumour-cell-aggregating potency of the absorbed and unabsorbed APFs of glycoprotein nature may be associated with the protein portions in their molecules, not with the carbohydrate portions, since the effects of these factors were similarly abolished by digestion with trypsin, but remained unchanged by oxidation with periodate. It has been demonstrated that proteins may play a key role in the aggregating effect of sea sponge extract (Gasic and Galanti, 1966) of purified aggregation factor of siliceous sponge (Müller *et al.*, 1976) and of porcine thyroid cell extract (Giraud *et al.*, 1974). It is thus assumed that the aggregating effect of the absorbed and unabsorbed APFs may be initiated by interaction of the protein portions in their molecules with some components at the surface of the tumour cells.

The present findings suggest that tumour-cell aggregation by the unabsorbed APF may be a consequence of the interaction of the protein structure in this APF molecule with certain carbohydrate molecules such as α-methyl-D-mannoside or D-mannose at the tumour-cell surface, because the potency of the APF was inhibited specifically by these carbohydrates. On the other hand, it is supposed that tumour-cell aggregation by the absorbed APF may be initiated by interaction of the protein structure in this APF molecule with certain carbohydrate molecules such as N-acetyl-D-glucosamine at the tumour-cell surface, because the activity of the APF was inhibited specifically by this carbohydrate or ovomucoid.

It is of special interest that these APFs may respectively bind with specific carbohydrates resulting in the complete inhibition of the potency. It has been widely known that the interactions of lectins with cells can, in many instances, be inhibited specifically by simple sugars (Mäkelä, 1957; Goldstein *et al.*, 1965; Smith and Goldstein, 1967; Sharon and Lis, 1972); this has led to the conclusion that lectins bind specifically to saccharides on the surface of the cells. Accordingly, it is suggested that the absorbed and unabsorbed APFs may bind with different saccharide sites at the tumour-cell surface for induction of cell aggregation. However, such a conclusion should await the separation of the receptor substances specific for the absorbed or unabsorbed APF from the tumour–cell surface, and the chemical analysis of the saccharides.

The present results also demonstrate a functional difference between the unabsorbed and absorbed APFs for induction of binding structures in AH109A cells. The unabsorbed APF induced cell aggregation (in the form of simple apposition) and then developed well-defined tripartite junctional complexes, including intermediate junctions, desmosomes and focal tight junctions during a period of 24 h incubation; the electronmicroscopic features closely resembled those induced by a mixture of the unabsorbed and absorbed APFs (Ishimaru *et al.*, 1975, 1976). On the other hand, the absorbed APF at a similar activity produced simple apposition with no development of intermediate junctions, desmosomes or focal tight junctions. Accordingly, it seemed reasonable that development by a mixture of these APFs of tripartite junctional complexes in the adherent cells may be associated with the function of the unabsorbed APF.

We would like to record our appreciation to Dr R. Kurano and Dr S. Tokuda for their technical cooperation. This work was supported in part by a special grant for cancer research from the Japanese Ministry of Education, Science and Culture.

### REFERENCES

Aub, J. C., Sanford, B. H. & Cote, M. N. (1965) Studies on Reactivity of Tumour and Normal Cells to a Wheat Germ Agglutinin. *Proc. natn. Acad. Sci. U.S.A.*, **54**, 396.

Axén, R., Porath, J. & Ernback, S. (1967) Chemical Coupling of Peptides and Proteins to Polysaccharides by Means of Cyanogen Halides. *Nature, Lond.*, **214**, 1302.

Biddle, F., Cronin, A. P. & Sanders, F. K. (1970) The Interaction between Wheat Germ Agglutinin and Receptors on Normal and Transformed Cells and on Erythrocytes. *Cytobios*, **5**, 9.

Burger, M. M. (1969) A Difference in the Architecture of the Surface Membrane of Normal and Virally Transformed Cells. *Proc. natn. Acad. Sci. U.S.A.*, **62**, 994.

Burger, M. M. & Goldberg, A. R. (1967) Identification of a Tumour-specific Determinant on Neoplastic Cell Surfaces. *Proc. natn. Acad. Sci. U.S.A.*, **57**, 359.

Farquhar, M. G. & Palade, G. E. (1963) Junctional Complexes in Various Epithelia. *J. Cell Biol.*, **47**, 197.

Gasic, G. J. & Galanti, N. L. (1966) Proteins and Disulfide Groups in the Aggregation of Dissociated Cells of Sea Sponges. *Science, N.Y.*, **151**, 203.

Giraud, A., Fayet, G. & Lissitzky, S. (1974) Thyrotropin-induced Aggregation-promoting Factors of Adult Cultured Thyroid Cells. *Expl Cell Res.*, **87**, 359.

Goldstein, I. J., Hollerman, C. E. & Smith, E. E. (1965) Protein–Carbohydrate Interaction. II. Inhibition Studies on the Interaction of Concanavalin A with Polysaccharides. *Biochemistry*, **4**, 876.

Inbar, M. & Sachs, L. (1969) Structural Difference in Sites on the Surface Membrane of Normal and Transformed Cells. *Nature, Lond.*, **223**, 710.

Ishimaru, Y., Ishihara, H. & Hayashi, H. (1975) An Electron Microscopic Study of Tumour Cell Adhesiveness Induced by Aggregation Promoting Factor from Rat Ascites Hepatoma Cells. *Br. J. Cancer*, **31**, 207.

Ishimaru, Y., Kudo, K., Ishihara, H. & Hayashi, H. (1976) The Induction of Tumour Cell Adhesiveness and Intercellular Junctions by a Glycoprotein of Rat Ascites Hepatoma Cell Surface. *Br. J. Cancer*, **34**, 426.

Kudo, K., Hanaoka, Y. & Hayashi, H. (1976) Characterization of Tumour Cell Aggregation Promoting Factor from Rat Ascites Hepatoma Cells: Separation of Two Factors with Different Antigenic Property. *Br. J. Cancer*, **33**, 79.

Kudo, K., Tasaki, I., Hanaoka, Y. & Hayashi, H. (1974) A Tumour Cell Aggregation Promoting Substance from Rat Ascites Hepatoma Cells. *Br. J. Cancer*, **30**, 549.

Lentz, T. L. & Trinkaus, J. P. (1971) Differentiation of the Junctional Complex of Surface Cells in the Developing Fundulus Blastoderm. *J. Cell Biol.*, **48**, 455.

Lis, H., Sela, B. A., Sachs, L. & Sharon, N. (1970) Specific Inhibition by N-acetyl-D-galactosamine of the Interaction between Soybean Agglutinin and Animal Cell Surfaces. *Biochim. biophys. Acta*, **211**, 582.

Lowry, O. H., Rosebrough, N. J., Farr, A. L. & Randall, R. J. (1951) Protein Measurement with the Folin Phenol Reagent. *J. biol. Chem.*, **193**, 265.

Mäkelä, O. (1957) Studies in Hemagglutinins of Leguminosae Seeds. *Ann. Med. exp. Biol. Fenn.*, **35**, Suppl. 11.

Matsumoto, I. & Osawa, T. (1972) The Specific Purification of Various Carbohydrate-binding

Hemagglutinins. *Biochem. biophys. Res. Commun.*, **46**, 1810.

Müller, W. E. G., Müller, I., Kurelec, B. & Zahn, R. K. (1976) Species-specific Aggregation Factor in Sponges. IV. Inactivation of the Aggregation Factor by Mucoid Cells from Another Species. *Expl Cell Res.*, **98**, 31.

Pessac, B. & Defendi, V. (1971) Cell Aggregation: Role of Acid Mucopolysaccharides. *Science, N.Y.*, **175**, 898.

Pessac, B. & Defendi, V. (1972) Evidence for Distinct Aggregation Factors and Receptors in Cells. *Nature, New Biol.*, **238**, 13.

Sharon, N. & Lis, H. (1972) Lectins: Cell-agglutinating and Sugar-specific Proteins. *Science, N.Y.*, **177**, 949.

Smith, E. E. & Goldstein, I. J. (1967) Protein–Carbohydrate Interaction. V. Further Inhibition Studies Directed toward Defining the stereochemical Requirements of the Reactive Sites of Concanavalin A. *Archs. Biochem. Biophys.*, **121**, 88.

Trelstad, R. L., Hay, E. D. & Revel, J. P. (1967) Cell Contact during Early Morphogenesis in the Chick Embryo. *Develop. Biol.*, **16**, 78.


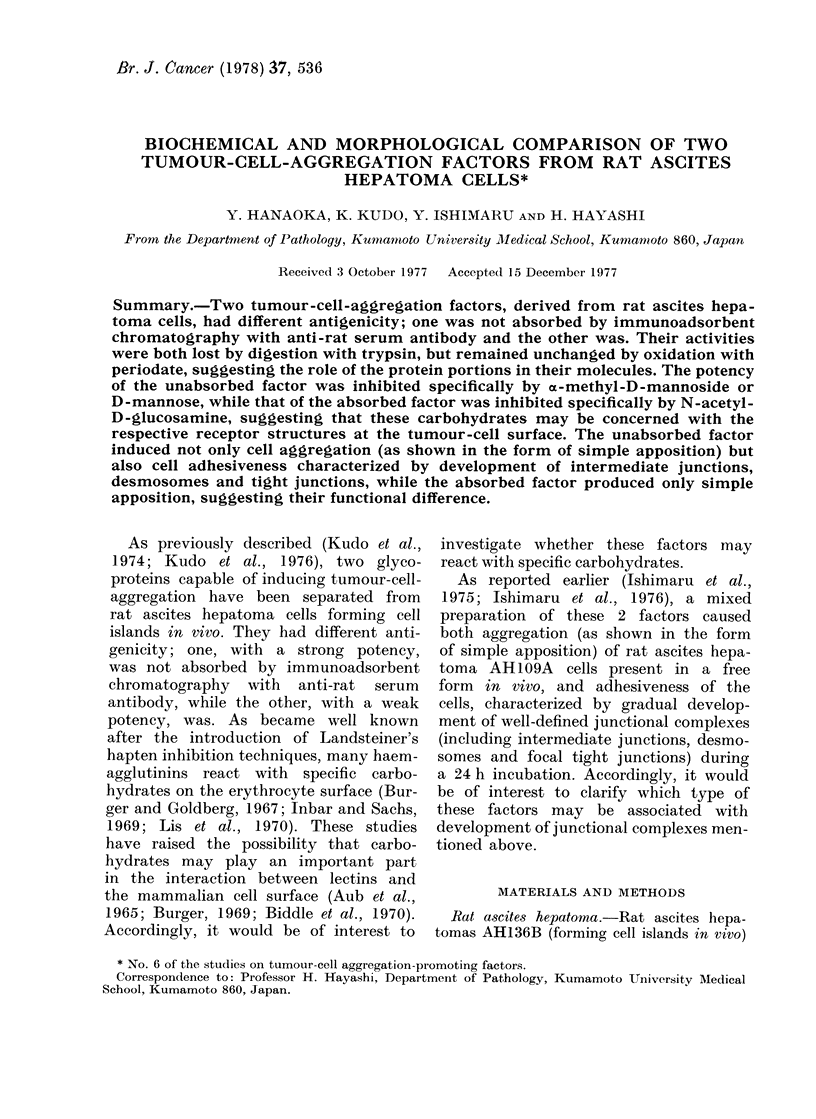

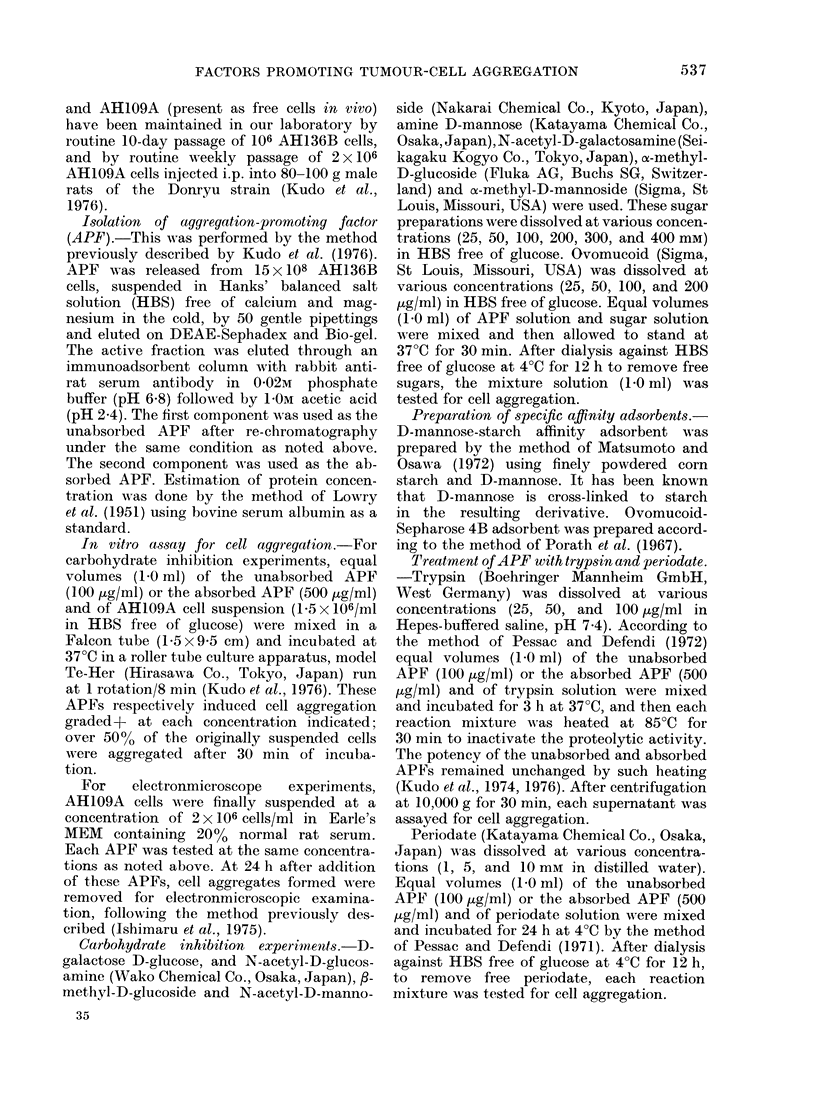

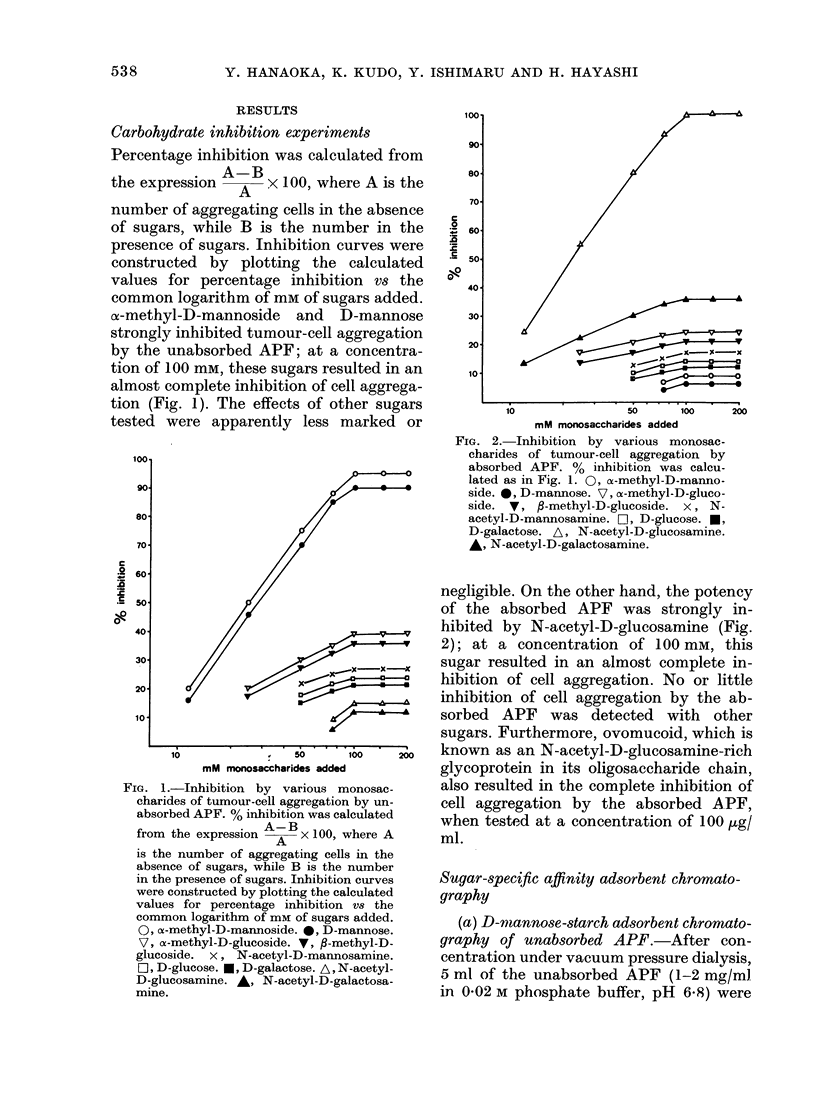

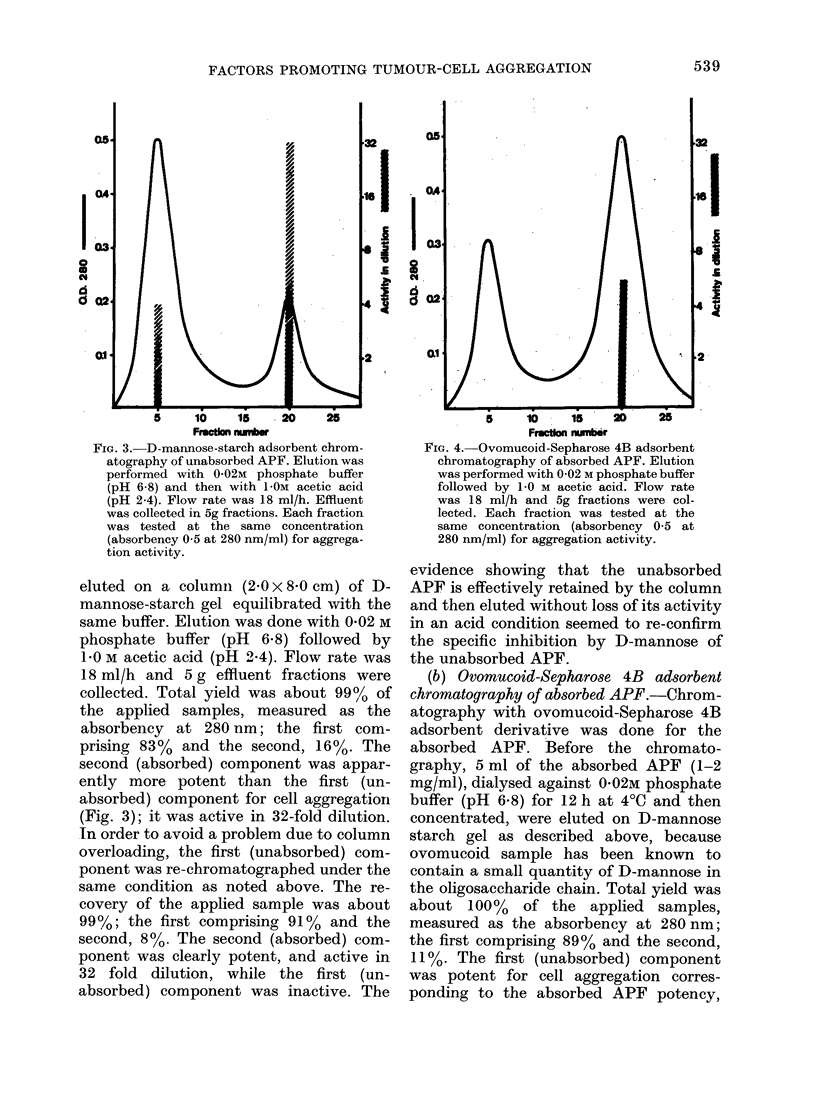

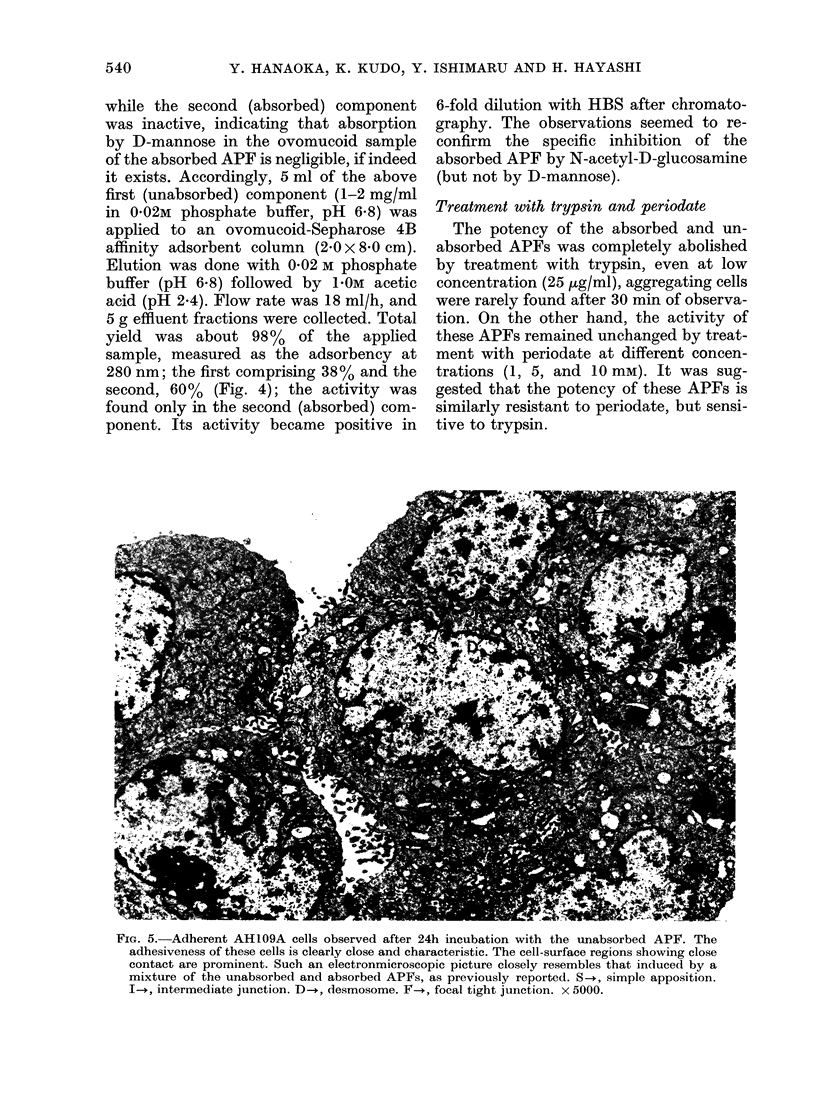

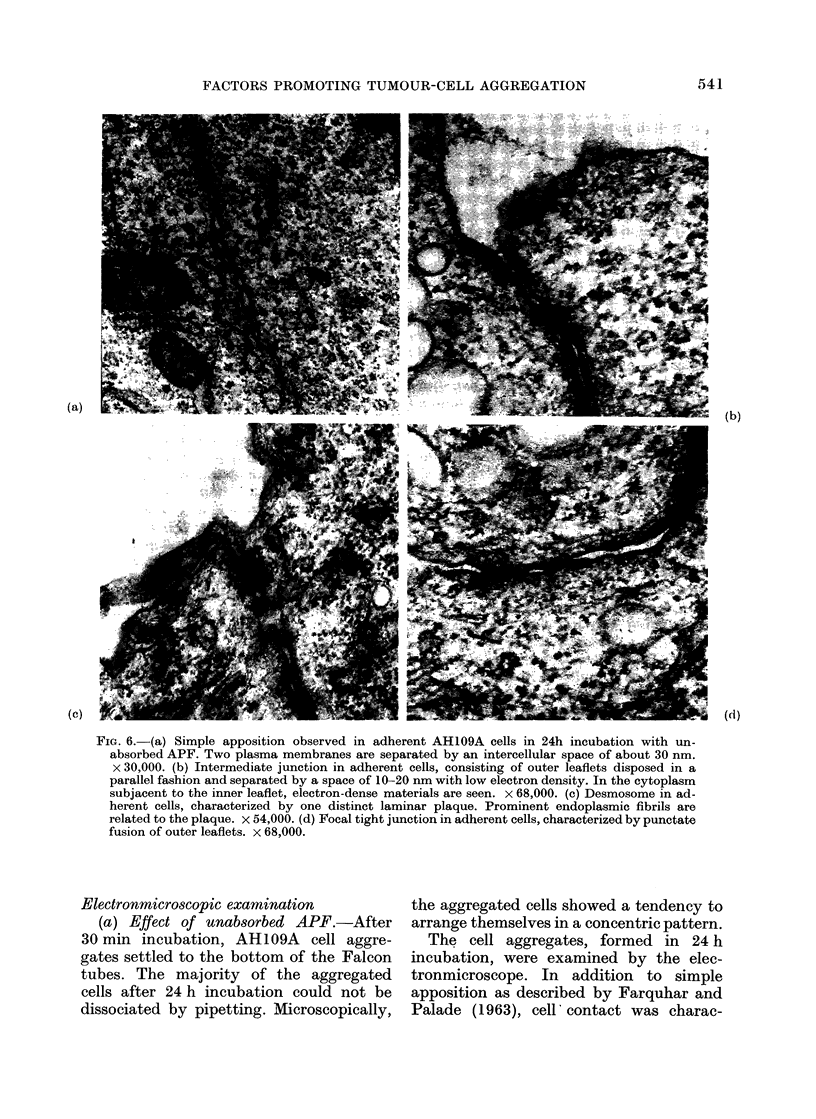

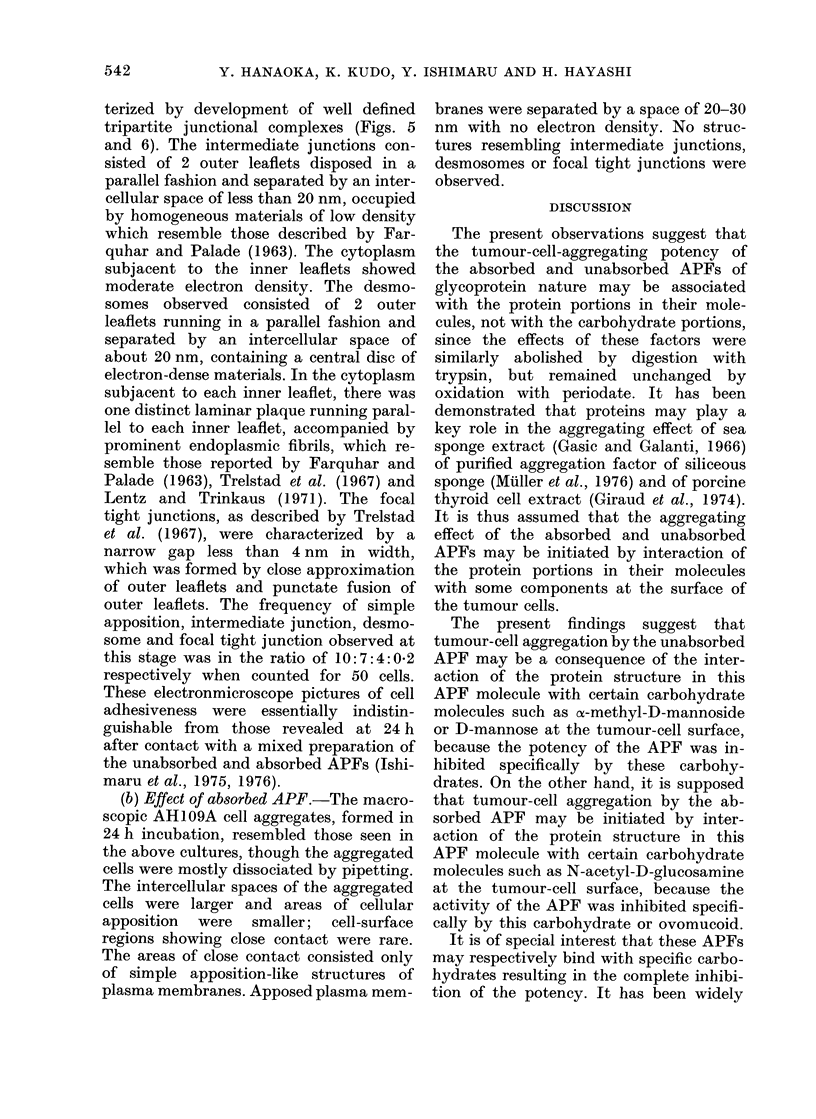

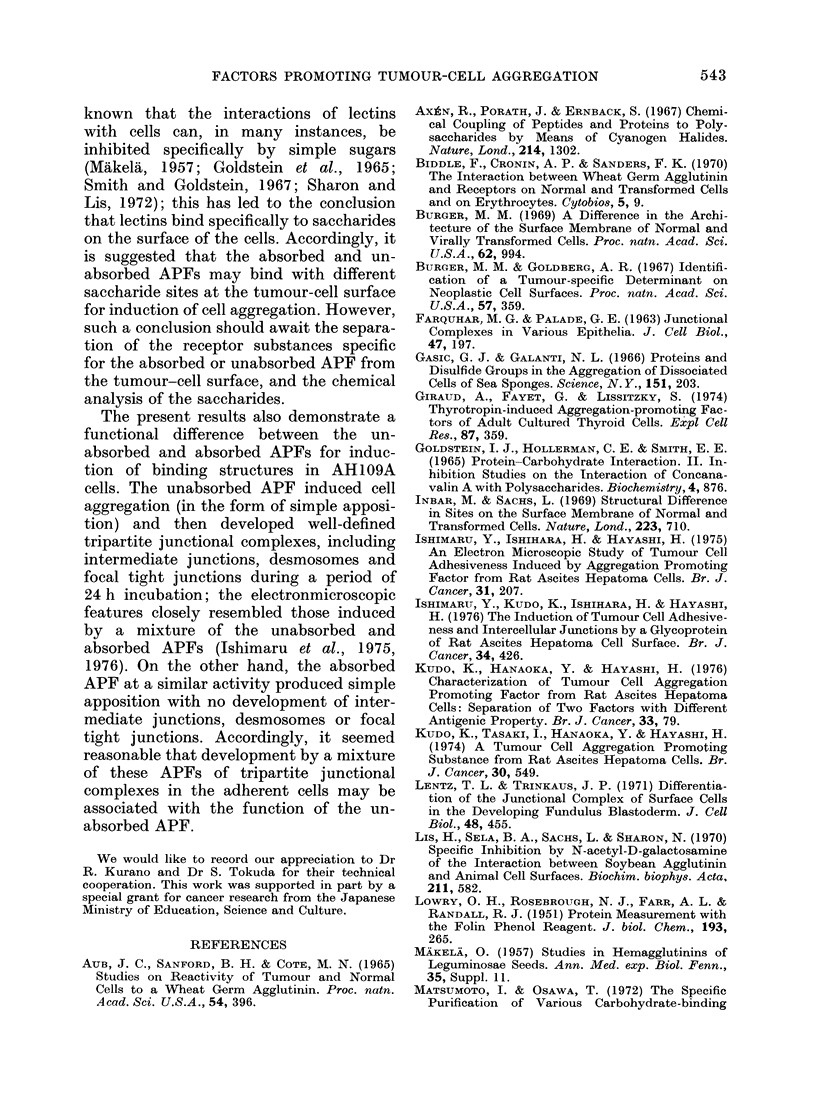

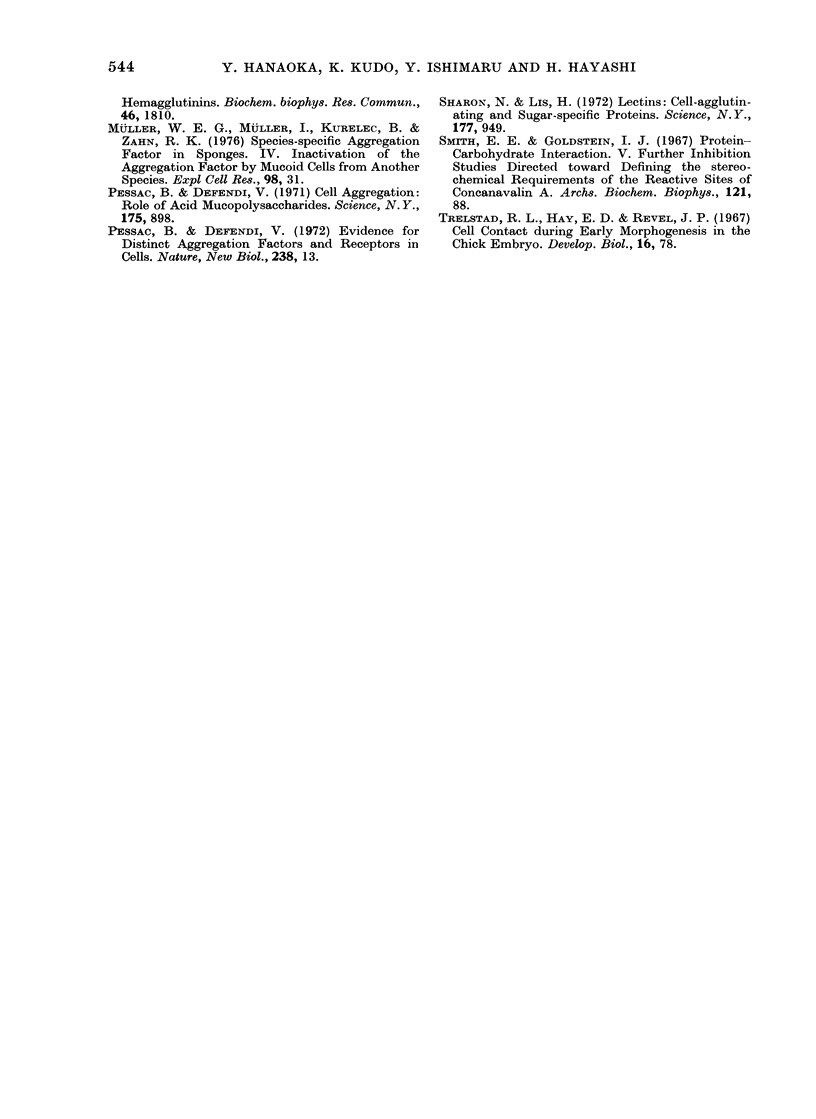


## References

[OCR_00709] Aub J. C., Sanford B. H., Cote M. N. (1965). Studies on reactivity of tumor and normal cells to a wheat germ agglutinin.. Proc Natl Acad Sci U S A.

[OCR_00715] Axén R., Porath J., Ernback S. (1967). Chemical coupling of peptides and proteins to polysaccharides by means of cyanogen halides.. Nature.

[OCR_00727] Burger M. M. (1969). A difference in the architecture of the surface membrane of normal and virally transformed cells.. Proc Natl Acad Sci U S A.

[OCR_00733] Burger M. M., Goldberg A. R. (1967). Identification of a tumor-specific determinant on neoplastic cell surfaces.. Proc Natl Acad Sci U S A.

[OCR_00755] GOLDSTEIN I. J., HOLLERMAN C. E., SMITH E. E. (1965). PROTEIN-CARBOHYDRATE INTERACTION. II. INHIBITION STUDIES ON THE INTERACTION OF CONCANAVALIN A WITH POLYSACCHARIDES.. Biochemistry.

[OCR_00744] Gasic G. J., Galanti N. L. (1966). Proteins and disulfide groups in the aggregation of dissociated cells of sea sponges.. Science.

[OCR_00749] Giraud A., Fayet G., Lissitzky S. (1974). Thyrotropin-induced aggregation-promoting factors of adult cultured thyroid cells.. Exp Cell Res.

[OCR_00760] Inbar M., Sachs L. (1969). Structural difference in sites on the surface membrane of normal and transformed cells.. Nature.

[OCR_00765] Ishimaru Y., Ishihara H., Hayashi H. (1975). An electron microscopic study of tumour cell adhesiveness induced by aggregation promoting factor from rat ascites hepatoma cells.. Br J Cancer.

[OCR_00772] Ishimaru Y., Kudo K., Ishihara H., Hayashi H. (1976). The induction of tumour cell adhesiveness and intercellular junctions by a glycoprotein of rat ascites hepatoma cell surface.. Br J Cancer.

[OCR_00779] Kudo K., Hanaoka Y., Hayashi H. (1976). Characterization of tumour cell aggregation promoting factor from rat ascites hepatoma cells: Separation of two factors with different antigenic property.. Br J Cancer.

[OCR_00786] Kudo K., Tasaki I., Hanaoka Y., Hayashi H. (1974). A tumour cell aggregation promoting substance from rat ascites hepatoma cells.. Br J Cancer.

[OCR_00805] LOWRY O. H., ROSEBROUGH N. J., FARR A. L., RANDALL R. J. (1951). Protein measurement with the Folin phenol reagent.. J Biol Chem.

[OCR_00792] Lentz T. L., Trinkaus J. P. (1971). Differentiation of the junctional complex of surface cells in the developing Fundulus blastoderm.. J Cell Biol.

[OCR_00798] Lis H., Sela B. A., Sachs L., Sharon N. (1970). Specific inhibition by N-acetyl-D-galactosamine of the interaction between soybean agglutinin and animal cell surfaces.. Biochim Biophys Acta.

[OCR_00816] Matsumoto I., Osawa T. (1972). The specific purification of various carbohydrate-binding hemagglutinins.. Biochem Biophys Res Commun.

[OCR_00825] Müller W. E., Müller I., Kurelec B., Zahn R. K. (1976). Species-specific aggregation factor in sponges. IV. Inactivation of the aggregation factor by mucoid cells from another species.. Exp Cell Res.

[OCR_00832] Pessac B., Defendi V. (1972). Cell aggregation: role of acid mucopolysaccharides.. Science.

[OCR_00842] Sharon N., Lis H. (1972). Lectins: cell-agglutinating and sugar-specific proteins.. Science.

[OCR_00847] Smith E. E., Goldstein I. J. (1967). Protein-carbohydrate interaction. V. Further inhibition studies directed toward defining the stereochemical requirements of the reactive sites of concanavalin A.. Arch Biochem Biophys.

[OCR_00855] Trelstad R. L., Hay E. D., Revel J. D. (1967). Cell contact during early morphogenesis in the chick embryo.. Dev Biol.

